# Characteristics of Patients with Mild to Moderate Primary Pulmonary Coccidioidomycosis

**DOI:** 10.3201/eid2006.131842

**Published:** 2014-06

**Authors:** Janis E. Blair, Yu-Hui H. Chang, Meng-Ru Cheng, Laszlo T. Vaszar, Holenarasipur R. Vikram, Robert Orenstein, Shimon Kusne, Stanford Ho, Maria T. Seville, James M. Parish

**Affiliations:** Mayo Clinic Hospital, Phoenix, Arizona, USA (J.E. Blair, H.R. Vikram, R. Orenstein, S. Kusne, M.T. Seville);; Mayo Clinic, Scottsdale, Arizona, USA (Y.-H. H. Chang, M.-R. Cheng, L.T. Vaszar, J.M. Parish);; Arizona State University, Tempe, Arizona, USA (S. Ho)

**Keywords:** acute pulmonary coccidioidomycosis, antifungal therapy, Coccidioides, coccidioidomycosis, community-acquired pneumonia, fungi

## Abstract

In Arizona, USA, primary pulmonary coccidioidomycosis accounts for 15%–29% of community-acquired pneumonia. To determine the evolution of symptoms and changes in laboratory values for patients with mild to moderate coccidioidomycosis during 2010–2012, we conducted a prospective 24-week study of patients with primary pulmonary coccidioidomycosis. Of the 36 patients, 16 (44%) were men and 33 (92%) were White. Median age was 53 years, and 20 (56%) had received antifungal treatment at baseline. Symptom scores were higher for patients who received treatment than for those who did not. Median times from symptom onset to 50% reduction and to complete resolution for patients in treatment and nontreatment groups were 9.9 and 9.1 weeks, and 18.7 and 17.8 weeks, respectively. Median times to full return to work were 8.4 and 5.7 weeks, respectively. One patient who received treatment experienced disseminated infection. For otherwise healthy adults with acute coccidioidomycosis, convalescence was prolonged, regardless of whether they received antifungal treatment.

## Abstract 2

Convalescence is prolonged, regardless of whether the patient receives treatment.

Coccidioidomycosis is a fungal infection caused by fungi of the genus *Coccidioides.* This illness is endemic to the southwestern United States. An estimated 150,000 infections occur annually, ≈60% in Arizona (*1*). The incidence of infection in this coccidioidomycosis-endemic area has considerably increased from 5.3 cases per 100,000 population in 1998 to 42.6 cases per 100,000 population in 2011 (*2*). Every year, ≈3% of area inhabitants become infected (*3*) through inhalation of airborne arthroconidia (spores), which results in mild to severe febrile respiratory illness (*4*,*5*). Extrapulmonary infection occurs in 1%–5% of patients with symptomatic infections (*5*,*6*).

Among Arizona patients with community-acquired pneumonia, 15%–29% have primary pulmonary coccidioidomycosis (*7*–*9*). Differentiating coccidioidal infection from pneumonia caused by viruses or bacteria is difficult. However, unlike other causes of community-acquired pneumonia, coccidioidomycosis is characterized by slow resolution of symptoms and extreme fatigue (*10*,*11*).

Symptomatic primary pulmonary coccidioidomycosis can range from mild to severe. Severe coccidioidomycosis has been defined as infection requiring hospitalization (*12*–*14*). Little research has focused on milder symptomatic forms. Although mild to moderate infection has not been clearly defined, it is characterized by symptomatic illness that does not require patient hospitalization. In the study reported here, we sought to describe the clinical course of mild to moderate pulmonary coccidioidomycosis in patients who did or did not receive antifungal therapy.

## Methods

From March 1, 2010, through October 31, 2012, at Mayo Clinic in Scottsdale, Arizona, USA, we conducted a 24-week, prospective, observational study of patients with mild to moderate symptomatic primary coccidioidomycosis. Our goal was to describe the course of illness-related signs and symptoms, laboratory values, and radiographic findings. This study was approved by the Mayo Clinic Institutional Review Board and included only those patients who had previously consented to the use of their medical records for research purposes. To be eligible, patients must have been >18 years of age, had primary pulmonary coccidioidomycosis, been symptomatic for <2 months, and had >2 signs or symptoms at enrollment. Signs and symptoms included (but were not limited to) fever, chills, night sweats, headache, joint aches, muscle pains, cough, rash, fatigue, inspiratory chest pain, and shortness of breath. The diagnosis was either confirmed (according to positive culture results or histologic findings) or probable (according to typical symptoms and radiographic abnormalities, with positive serologic test results). Also for patient eligibility, serologic test results were required to be positive for IgG against *Coccidioides* spp. by enzyme immunoassay (Meridian Bioscience, Inc., Cincinnati, OH, USA), immunodiffusion, or complement fixation, or for IgM by immunodiffusion. No remuneration or other incentive was provided for study participation. Exclusion criteria were as follows: hospitalization, clinical evidence of overtly extrathoracic coccidioidomycosis, laboratory or radiographic findings of severe or disseminated infection (e.g., an initial complement fixation titer >1:32, chest radiographic abnormalities with miliary distribution, lung involvement >50%, or large pleural effusion), concurrent conditions associated with increased risk for severe or disseminated coccidioidomycosis (e.g., any viral load of HIV, chemotherapy within 6 months for cancer, solid organ or hematologic transplantation, hematologic malignancy [active or remote], diabetes mellitus, or pregnancy), receipt of immunosuppressive medications (e.g., tumor necrosis factor inhibitors, calcineurin inhibitors, mycophenolate mofetil, sirolimus, or chronic oral corticosteroids [equivalent dose of >5 mg/day, excluding inhaled, topical, or limited and transient oral corticosteroids for <5 days]), concurrent cardiopulmonary conditions (e.g., pulmonary coinfection, asthma or chronic obstructive pulmonary disease, cardiomyopathy), or underlying liver disease or stage 4 or 5 kidney disease (glomerular filtration rate <29 mL/min/1.73 m^2^).

Treatment decisions were determined by the treating physicians, and whether a patient received antifungal therapy before enrollment was recorded (medication name, dose, frequency, duration). After patients were enrolled, physician investigators determined the need to initiate or continue antifungal medication on a case-by-case basis. Patients were assigned to the treatment group if at any time before enrollment through study completion they received any antifungal treatment.

The medical care for coccidioidomycosis was provided by physician investigators and conducted in a standardized fashion. The initial evaluation included a complete blood cell count, comprehensive metabolic panel, serologic testing for HIV, pregnancy testing, and serologic testing for coccidioidomycosis (by enzyme immunoassay, immunodiffusion, and complement fixation); collection of microbiological specimens, if applicable (mostly sputum for fungal culture); and analysis of chest radiographs. Patients were evaluated clinically, serologically, and radiographically (chest) at enrollment and at 4, 12, 16, and 24 weeks.

Signs and symptoms were assessed by using modified standardized Mycosis Study Group symptom scores previously used in coccidioidomycosis clinical trials (*15*–*17*). Symptom scores were based on answers to a questionnaire listing common symptoms of coccidioidomycosis (fever [subjective or measured], chills, night sweats, headache, joint aches, muscle pain, rash, fatigue, anorexia or weight loss, swelling, cough, shortness of breath, pain during inspiration, hemoptysis). Additional symptoms noted by patients at enrollment were added to the list. Each symptom was scored as 1 point, and points were tallied for an enrollment symptom score. Each time signs and symptoms were assessed, patients were directly asked about symptom presence or absence within the preceding week. Symptoms could be added to the score as the course of illness progressed. After patient enrollment, symptoms were tallied weekly. When this score declined to 50% of the enrollment score for 2 consecutive weeks, symptoms were assessed every 2 weeks for 2 episodes, then monthly for 6-month follow-up visits. Baseline and monthly fatigue levels were assessed by using the fatigue severity scale (*10*,*18*), and health-related quality of life was assessed by using the 36-Item Short Form Health Survey (*19*). Full-time or part-time attendance at work or school was recorded during assessments. The coccidioidal radiology score was based on Mycosis Study Group scoring (*15*,*16*) as follows: size (1 point for lesions <5.0 cm; 2 for >5.0 cm), spread (1 point if unilateral; 2 if bilateral), and other characteristics (1 point each for pulmonary cavity, hilar lymphadenopathy, or pleural effusion).

All end points were established a priori. The primary end point was time required to achieve a 50% decrease in symptom score. Secondary end points included time to 100% symptom resolution (excluding fatigue), time to resumption of all activities of daily living, time to achieve 50% and 100% reductions in fatigue score, time to 50% reduction in Mycosis Study Group score, time to full attendance at work or school, and time to 50% improvement in quality-of-life score. Secondary end points included comparison of end points between patients in the treatment and nontreatment groups. All recorded times were normalized to time of symptom onset rather than time from enrollment.

Patient characteristics and the occurrence of symptoms were summarized as counts and percentages and were compared between treatment groups by using the χ^2^ test or the Fisher exact test. The total symptom score, time to resolution, and quality-of-life summary scores were summarized as medians and interquartile ranges and were compared by using the Wilcoxon rank–sum test. The prevalence of each symptom over time was plotted. The prevalence of each symptom was modeled by the generalized estimating equation, and the difference of the prevalence between time points was evaluated statistically. All analyses were performed by using SAS 9.2 (SAS Institute Inc., Cary, NC, USA). All hypothesis tests were 2 sided, and statistical significance was defined as p<0.05.

## Results

**Figure 1:**
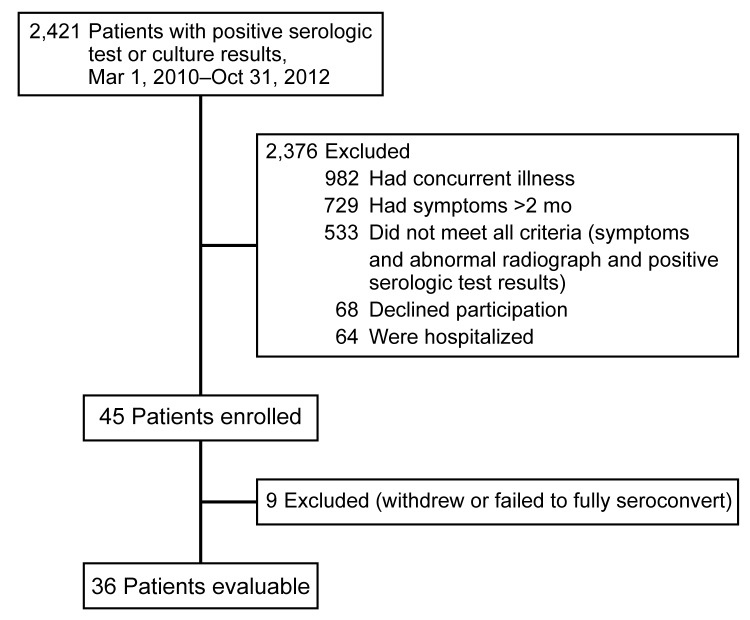
Coccidioidomycosis patient enrollment and exclusions, Arizona, USA, March 1, 2010–October 31, 2012.

During the study period, 45 patients with primary pulmonary coccidioidomycosis were enrolled; 9 withdrew consent or did not fully seroconvert, leaving 36 with probable infection for inclusion in the study (Figure 1). Of these, 27 (75%) patients remained in the study through week 24 of their illness, and 20 (56%) completed the entire 24 weeks of observation after enrollment. Median time from symptom onset to enrollment was 33 days.

At the time of enrollment, 16 (44%) patients had not received antifungal therapy (Table 1). Among the 20 (56%) patients who had received antifungal therapy, treatment was initiated by nonstudy medical practitioners before enrollment for 17 and by study physicians on the day of enrollment for 3. Antifungal treatment was initiated at a median of 21 days of symptoms (range 4–46 days after onset [interquartile range 11–32 days]). Of the 20 who received treatment, 18 received fluconazole at 400 mg per day for a median of 8.5 weeks (range 1.5–28.0 weeks). The median weight of patients in the treatment group was 84.8 kg (interquartile range 73–91 kg). Twenty-six patients (16 treatment, 10 nontreatment) had received empiric treatment with 1 or 2 courses of antibacterial drugs before their coccidioidomycosis diagnosis.

**Table 1:** Characteristics of 36 patients with primary pulmonary coccidioidomycosis, Arizona, USA, March 1, 2010–October 31, 2012*

Characteristic	Total, N = 36	Antifungal treatment		p value
		Yes, n = 20	No, n = 16	
Sex, no. (%)				
M	16 (44)	11 (55)	5 (31)	0.15†
F	20 (56)	9 (45)	11 (69)	0.15†
Age, y, median (range)	53 (21–79)	52 (28–79)	53 (21–68)	0.48‡
Race/ethnicity, no. (%)				0.65†
White	33 (92)§	18 (90)	15 (94)	
Hispanic	1 (3)	1 (5)	0	
Asian	2 (6)	1 (5)	1 (6)	
Follow-up time, median (IQR), wk	24 (24.0–24.0)	24 (21.5–24.0)	24 (24.0–24.0)	
Concurrent illness¶				
Rheumatologic, no. (%)#	1 (3)	1 (5)	0	0.35†
Prior remote cancer, no recurrence, no./total (%)#	3/35 (9)	2/19 (11)	1 (6)	0.65†
Employment				
Employed at time of coccidioidomycosis, no. (%)	22 (61)	14 (70)	8 (50)	0.22†
Illness resulted in work absences, no. (%)	18/22 (82)	12/14 (86)	6/8 (75)	0.53†
Days absent, median no. (IQR), [range]	10 (5–14) [1–28]	10 (5–15) [2–28]	7 (4–12) [1–13]	0.32‡
School attendance				
Attending at time of coccidioidomycosis, no. (%)	3 (8)	2 (10)	1 (6)	0.68
Illness resulted in absences, no./total (%)	1/3 (33)	0	1/1 (100)	0.08
Coccidioidal symptoms, ever present, no. (%)				
Fatigue	36 (100)	20 (100)	16 (100)	>0.99†
Fever	31 (86)	19 (95)	12 (75)	0.08†
Chills	32 (89)	19 (95)	13 (81)	0.19†
Cough	34 (94)	18 (90)	16 (100)	0.19†
Night sweats	29 (81)	17 (85)	12 (75)	0.45†
Headache	29 (81)	16 (80)	13 (81)	0.93†
Chest pain	25 (69)	15 (75)	10 (62)	0.52†
Arthralgia	25 (69)	13 (65)	12 (75)	0.59†
Rash	23 (64)	12 (60)	11 (69)	0.58†
Coccidioidal symptoms score at enrollment, median (IQR)	5.0 (3.0–7.0)	5.5 (5.0–7.5)	4.0 (3.0–5.0)	0.02‡
Diagnostic test results				
Median chest radiograph score at enrollment	2.0	2.0	2.0	0.72‡
Positive serologic results at enrollment, no./total (%)				
EIA IgM	34/35 (97)	18/19 (95)	16/16 (100)	0.35†
EIA IgG	26/35 (74)	12/19 (63)	14/16 (88)	0.1†
ID IgM	14/36 (39)	7/20 (35)	7/16 (44)	0.59†
ID IgG	19/35 (53)	10/20 (50)	9/16 (56)	0.71†
Positive IgG by CF	12/32 (38)	6/16 (38)	6/16 (38)	>0.99†

*IQR, interquartile range; EIA, enzyme immunoassay; ID, immunodiffusion; CF, complement fixation. †By χ^2^ test. ‡By Wilcoxon rank-sum test. §95% CI for White race was 77.5%–98.2%. ¶No patients had pulmonary, cardiovascular, kidney, or liver disease. #Patient was not receiving any immunosuppressive treatment or chemotherapy.

At enrollment, ongoing fever was more common among patients who had received treatment than among those who had not (8/20 [40%] vs. 1/16 [6.2%], respectively; p = 0.02), although other symptoms did not differ by group (Table 1). At enrollment, symptom scores were higher among patients in the treatment than in the nontreatment group (median 5.5 vs. 4.0, respectively; p = 0.02).

In terms of occupation, 22 patients were employed and 3 were full-time students (Table 1). The median number of whole workdays missed was 10 (range 1–28 days). One student missed 10 days of school.

At enrollment, no significant differences in serologic or radiographic findings were noted among patients in the treatment and nontreatment groups (Table 1). The percentages of patients with detectable complement fixation antibody at enrollment and at 4, 12, and 24 weeks were 38% (12/32), 61% (17/28), 59% (16/27), and 23% (5/22), respectively. Peak complement fixation titers (range 1:2–1:32) occurred 4 weeks after enrollment. Radiographic scores did not differ by group; and for most patients, a unilateral radiographic abnormality <5 cm was seen. Although radiographic abnormalities improved over time, abnormalities on chest radiographs, as reflected in median scores, did not decline from 2.0 at enrollment. At 24 weeks, one-half of the patients had residual granuloma.

Table 2 summarizes primary and secondary end points of the study and shows that times to most end points were similar for both groups; for the nontreatment and the treatment groups, the median times to 50% and 100% resolution of symptoms were 9.1 and 9.9 weeks and 17.8 and 18.7 weeks, respectively. The median times to 50% resolution of fatigue were 9.8 and 12.9 weeks, respectively. Of 27 patients, 13 (48%) indicated continued fatigue by week 24. Patients in the nontreatment group returned to full-time work sooner than did those in the treatment group (5.7 vs. 8.4 weeks, respectively) (p = 0.02).

**Table 2:** Comparison of onset of symptoms to time to study end points among 36 patients with primary pulmonary coccidioidomycosis, Arizona, USA, March 1, 2010– October 31, 2012*

Study end point		Antifungal treatment			p value
		Yes, n = 20	No, n = 16		
Time to 50% reduction in symptom score, wk					0.84†
Median		9.9	9.1		
Q1, Q3		7.0, 13.4	7.4, 17.1		
Range		4.0–24.0	3.7–24.4		
Time to complete symptom resolution, wk					0.65‡
Median		18.7	17.8		
Q1, Q3		13.6, 25.0	12.1, 24.0		
Range		8.9–29.6	8.7–27.1		
Time to 50% reduction in fatigue, wk§					0.59‡
Median		12.9	9.8		
Q1, Q3		8.0, 16.0	8.4, 15.9		
Range		5.0–29.6	4.4–25.6		
Time to full attendance at work, wk					0.02†
Median		8.4	5.7		
Q1, Q3		6.7, 14.8	5.1, 6.0		
Range		9.0–29.6	2.4–7.6		
Time to full attendance at school, wk					
Median			13.7		
Q1, Q3			13.7, 13.7		
Time to 50% improvement in PCS, wk					0.08‡
Median		20.4	13.8		
Q1, Q3		14.7, 26.0	10.4, 18.0		
Time to 50% improvement in MCS, wk					0.21‡
Median		12.0	8.1		
Q1, Q3		9.3, 14.7	6.6, 9.7		

*Q1, first quartile; Q3, third quartile; PCS, physical activity score as measured on the SF-36 General Health Survey; MCS, mental activity score as measured on the SF-36 General Health Survey. †By the Wilcoxon rank–sum test. ‡By the equal variance *t* test. §As reported on the Fatigue Severity Scale.

**Figure 2:**
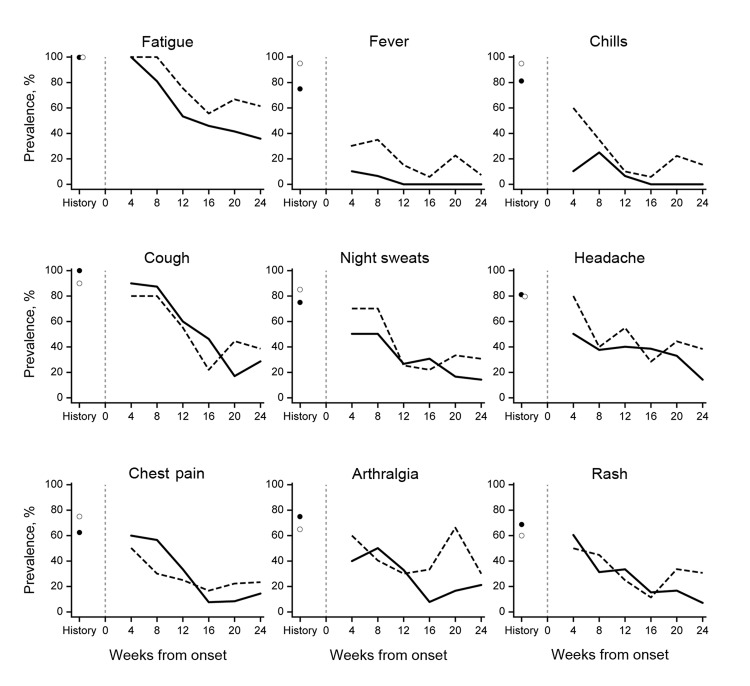
Presence of coccidioidomycosis symptoms from time of symptom onset, by treatment group, Arizona, USA, March 1, 2010–October 31, 2012. The graphs represent the percentages of patients who reported each symptom, from the time since onset of symptomatic illness. Solid lines and filled circles indicate the nontreatment group, and dashed lines and open circles indicate the treatment group. The vertical line indicates time of symptom onset. The circles to the left of the vertical line indicate the presence of symptoms at any time, including before study enrollment**.**

Figure 2 and Table 3 summarize symptom resolution for the 36 patients over time. Although symptom curves seemed to separate, especially from week 16 on, there was no statistical significance between these curves.

**Table 3:** Signs and symptoms since onset of coccidioidal Illness, among 36 patients with primary pulmonary coccidioidomycosis, Arizona, USA, March 1, 2010–October 31, 2012*

Symptom*	No. (%) patients				
	Week 4, n = 20†	Week 8, n = 36	Week 12, n = 35	Week 16, n = 31	Week 24, n = 27
Fatigue‡	20 (100)	33 (92)	22 (66)	16 (52)	13 (48)
Fever	4 (25)	8 (22)	3 (9)	1 (3)	1 (4)
Chills	7 (35)	11 (31)	3 (9)	1 (3)	2 (7)
Cough	17 (85)	30 (83)	20 (57)	10 (32)	9 (33)
Night sweats	12 (60)	22 (61)	9 (26)	8 (26)	6 (22)
Headache	13 (65)	14 (39)	17 (49)	10 (32)	7 (26)
Chest pain	11 (55)	15 (42)	10 (29)	4 (13)	5 (19)
Arthralgia	10 (50)	16 (44)	11 (31)	7 (23)	7 (26)
Rash	11 (55)	14 (39)	10 (29)	4 (13)	5 (19)

*As reported by patients responding directly to a question about presence of the symptom. †Sixteen patients had not yet enrolled in the study by the fourth week of symptom onset. ‡By direct question for presence of fatigue, not by Fatigue Severity Score.

The course of convalescence was typical for 35 patients and atypical for 1 patient. This previously healthy 34-year-old White man was initially seen at an external institution for a 3-week history of fever, night sweats, dry cough, headache, and rash. Serologic test results were positive for *Coccidioides* spp. by enzyme immunoassay and immunodiffusion, and chest radiographs demonstrated a 3-cm nodular infiltrate; the physician prescribed a nonstandard antifungal regimen of ketoconazole at 400 mg/day. When the patient was referred to our institution (Mayo Clinic Hospital, Phoenix, AZ, USA) for possible study participation 10 days later, his symptoms were nearly resolved and ketoconazole was discontinued by the study physician. One week after enrollment, the patient experienced a severe headache; subsequent cerebrospinal fluid analysis was consistent with aseptic meningitis, presumed to be caused by Coccidioides. He was given fluconazole at 800 mg/day and promptly improved clinically. Lifelong treatment is anticipated.

## Discussion

Over the past 2 decades, the incidence of coccidioidomycosis has markedly risen in the disease-endemic area (*1*,*2*). Until recently, little research has characterized the course of uncomplicated symptomatic illness, although experienced clinicians have observed that affected patients eventually recover (*11*,*20*). Each year in the disease-endemic area, an estimated 3% of the population becomes infected (*3*); therefore, even if 60% of the infected population is asymptomatic, the potential number of patients who may become ill enough to be unable to perform daily activities or work is substantial. This study prospectively characterized the prolonged clinical course of patients with mild to moderate primary pulmonary coccidioidomycosis.

A 2007 survey found that patients with coccidioidomycosis undifferentiated by severity, status of dissemination, or duration (acute or chronic) recalled experiencing symptoms for a median of 120 days and missing 14 days of work or 9 days of school (*21*). The study was limited by its retrospective nature, relying only on the ability of patients to recall details of their illness from the previous year, and these self-reports were not correlated with severity of illness (mild vs. severe or pulmonary vs. disseminated). Other investigators have demonstrated a median time of 95–98 days (roughly 14 weeks) to 50% resolution of symptoms (*11*). Still others have found that fatigue is severe at baseline and 4 months later (*10*) and that >25% of otherwise healthy college students with primary coccidioidomycosis required medical care for at least 4 months (*22*). Although our study focused only on persons with mild to moderate infection and no substantial concurrent conditions, results are similar to those of previous studies (long duration of symptoms, fatigue, and illness caused by primary coccidioidal infection).

In our study, we noted a typical pattern of clinical resolution and resumption of normal activities. Fever and chills were relatively short-lived (days to weeks), but other symptoms, such as cough and fatigue, lasted weeks to months.

Figure 2 delineates resolution of symptoms over time among patients in the treatment and nontreatment groups. Statistical comparison of these curves did not identify any differences. For many symptoms, a bimodal curve appeared for the treatment group but not for the nontreatment group, suggesting that patients who received treatment experienced more symptoms in the second half of the observation period. However, when we fitted a longitudinal model to examine whether a difference existed between weeks 16 and 20 for each of the symptoms, no statistically significant differences were identified.

Most (82%) patients missed work for a median of 10 workdays. Those in the treatment group did not miss more workdays than those in the nontreatment group, but they did return to full-time employment more slowly (median 8.4 vs. 5.7 weeks). Whether this finding was the result of more severe illness in patients in the treatment group or other factors is not certain. However, this lost work productivity highlights the potentially profound economic cost of this illness in the coccidioidomycosis-endemic area.

Although the current study was strictly observational, we enrolled similar numbers of patients with similar demographic characteristics, regardless of treatment received. We classified the groups by any antifungal treatment and classified patients as having received treatment even if treatment was given for a short time or at a suboptimal dosage. Two patients received treatment for <1 month (1.5 weeks and 2 weeks), and another received nonstandard treatment at an external institution (ketoconazole at 400 mg/day). However, because our results showed no difference with and without inclusion of such patients (data not shown), these data did not influence our overall findings. Of 20 patients in the treatment group, 18 received fluconazole at 400 mg/day for a median duration of 8.5 weeks. For some patients, antifungal medications were discontinued because of medication intolerance; others initially received antifungal treatment from nonstudy medical providers and were subsequently determined by study physicians to not require treatment, which was then discontinued. The optimal duration of treatment for mild to moderate coccidioidomycosis has never been defined, and the fact that discontinuation of some antifungal medications occasionally preceded complete resolution of prolonged symptoms (e.g., occasional cough or prolonged fatigue) reflected the practices of our clinical investigators. Neither study design nor study power enabled identification of small differences in the clinical courses of disease among patients in the treatment versus nontreatment groups. However, we did not identify any clinical end points that showed a benefit to patients in the treatment group, who were statistically more symptomatic according to symptom score at study enrollment. Our results are similar to those of Ampel et al. (*11*), who found that patients in treatment and nontreatment groups reached 50% symptom reduction at the same time.

Patients in this study began receiving antifungal treatment at a median of 21 days from the onset of coccidioidomycosis symptoms. Most patients sought care from their medical providers within days of symptom onset, but when they had no clinical response to empirically prescribed antibacterial agents, further testing identified the coccidioidal etiologic agent of disease. Therefore, the delay in early treatment probably reflects the lack of recognition of coccidioidal illness (which causes nonspecific symptoms), the lack of an early and reliable diagnostic test, or both.

Although the Infectious Diseases Society of America treatment guidelines acknowledge differences of expert opinion regarding the need to treat primary coccidioidomycosis (*23*), the guidelines suggest identifying characteristics to facilitate diagnosis of moderate to severe infection in patients who might benefit from treatment (*23*). These guidelines recommend possible antifungal treatment for patients with symptoms lasting >2 months, night sweats >3 weeks, weight loss of >10%, inability to work, serologic complement fixation titer >1:16, bilateral infiltrates or involvement of at least one half of 1 lung, or prominent or persistent hilar adenopathy (*23*). In retrospect, many of the patients in our nontreatment group met >1 of these criteria, yet their illness resolved no more slowly than that of patients in the treatment group; this finding mandates further study to determine which patients with mild to moderate pulmonary coccidioidomycosis will benefit from antifungal treatment.

One challenge posed by previous and current studies is the lack of tests sensitive enough to identify coccidioidal infection early in its course. All current serologic tests take a few weeks to several weeks to show positivity (*24*). Because of potential diagnostic delays resulting from delayed seroconversion or the lack of clinical recognition of primary pulmonary coccidioidomycosis, patients received a diagnosis, received treatment, and were enrolled in the study at variable points relative to illness onset, which was typically 4–7 weeks. Therefore, the end points of the study were normalized to symptom onset.

Some potential participants were excluded from the study for lack of any positive serologic results other than detection of IgM by enzyme immunoassay. For some, an IgG response might have been inhibited by preexisting treatment with fluconazole, which has been reported (*25*). For others, detection of IgM by enzyme immunoassay might have been associated with false positivity, although published reports are divided on this point (*26*,*27*). Thus, in our effort to ensure that all study participants truly had coccidioidomycosis, we might have excluded patients with an incomplete serologic response.

Other limitations of this study are noteworthy. The study is small because we were able to identify and study only 36 patients within the given time frame. During enrollment, we recognized that some patients with typical signs and symptoms and coccidioidal seroconversion lacked identifiable abnormalities on chest radiographs early in their illness or had typical radiographic abnormalities but no definitive serologic test results. Thus, we probably excluded patients who had even milder forms of infection. Other enrollment difficulties included a delay to recognition and diagnosis beyond 2 months of illness, restrictive exclusion criteria, and the requirement for frequent follow-up visits at specific times over an extended period. Study dropout was a problem because the study was long, repetitive, and time intensive. Although most (27/36 [75%]) patients continued in the study through week 24 of their illness, 9 (25%) did not, which resulted in low numbers at the end of the study and reduced percentages of patients with various symptoms. More dropouts came from the treatment than the nontreatment group (7 vs. 2), and some patients dropped out before becoming asymptomatic. Last, if a patient’s symptoms had a different cause (e.g., viral bronchitis or fluconazole-related rash) during follow-up, their symptoms might have been misinterpreted by the patient and reported as coccidioidal symptoms; therefore, our results must be interpreted with caution.

Our study cohorts were selected for the absence of concurrent illnesses that might otherwise have affected manifestations or outcomes of coccidioidal illness, which might have given them the best possible course of illness resolution. We also enrolled primarily White patients (reflecting the 85% White [non-Hispanic] population of Maricopa County [*28*] and the proportion of White patients with coccidioidal infection reported to the Arizona Department of Health Services [82%] [*29*]); disseminated infection is generally less likely to develop in members of this group than in members of other racial or ethnic groups (e.g., Africans or Filipinos) (*30*). This narrow cohort limits the generalizability of our findings to other patient groups.

In conclusion, we believe that mild to moderate primary pulmonary coccidioidomycosis is a consequential illness that affects numerous persons residing in or traveling to the disease-endemic area. Our detailed description of patients with mild to moderate signs and symptoms of infection and the slow resolution of those signs and symptoms over time can better inform diagnosis, treatment, and prognosis for patients with coccidioidomycosis. Although we found no benefit from antifungal treatment, the study was neither designed nor powered to optimally address that issue. Given that the coccidioidal illness is substantial and prolonged, further study is warranted to optimally identify and treat this condition in such patients.
